# Montreal cognitive assessment as a cognitive outcome measure in progressive supranuclear palsy

**DOI:** 10.3389/fneur.2024.1501206

**Published:** 2024-12-04

**Authors:** Vanessa Ibrahim, Catherine Isroff, Christopher D. Stephen, Jay Iyer, Marian L. Dale, Douglas A. Gunzler, Ece Bayram, Tao Xie, Alex Pantelyat, Leila Montaser-Kouhsari, Indira Garcia-Cordero, Maria Carmela Tartaglia, Anthony E. Lang, Matthew Swan, Adam L. Boxer, Lawrence I. Golbe, Anne-Marie Wills

**Affiliations:** ^1^Department of Neurology, Massachusetts General Hospital, Harvard Medical School, Boston, MA, United States; ^2^Departments of Molecular and Cellular Biology and Statistics, Harvard University, Cambridge, MA, United States; ^3^Department of Neurology, Oregon Health & Science University, Portland, OR, United States; ^4^Population Health and Equity Research Institute, Center for Health Care Research and Policy, Case Western Reserve University at MetroHealth Medical Center, Cleveland, OH, United States; ^5^Parkinson and Other Movement Disorders Center, Department of Neurosciences, UC San Diego, La Jolla, CA, United States; ^6^Movement Disorder Program, Department of Neurology, University of Chicago Medicine, Chicago, IL, United States; ^7^Department of Neurology, Johns Hopkins University School of Medicine, Baltimore, MD, United States; ^8^Department of Neurology, Brigham and Women’s Hospital, Harvard Medical School, Boston, MA, United States; ^9^Tanz Centre for Research in Neurodegenerative Diseases, University of Toronto, Toronto, ON, Canada; ^10^Krembil Brain Institute, University Health Network, Toronto, ON, Canada; ^11^Edmond J. Safra Program in Parkinson's Disease, Rossy PSP Centre, Morton and Gloria Shulman Movement Disorders Clinic, Krembil Brain Institute, Toronto Western Hospital, University Health Network and Division of Neurology, University of Toronto, ON, Canada; ^12^Department of Neurology, Mount Sinai Beth Israel, Icahn School of Medicine at Mount Sinai, New York, NY, United States; ^13^Memory and Aging Center, Department of Neurology, University of California, San Francisco, San Francisco, CA, United States; ^14^Rutgers Robert Wood Johnson Medical School, New Brunswick, NJ, United States

**Keywords:** MoCA = Montreal Cognitive Assessment, PSP, cognitive outcome measure, fluency, progressive supranucelar palsy

## Abstract

**Background:**

The Montreal Cognitive assessment (MoCA) is a well-validated global cognitive screening instrument. Its validity in progressive supranuclear palsy (PSP) has not been assessed.

**Objectives:**

To evaluate the MoCA as an outcome measure in PSP clinical trials.

**Methods:**

Cognitive data from 162 participants in the placebo arm of the Biogen PASSPORT study (NCT03068468) were analyzed using linear mixed-effects modeling (LMM) and repeated measures correlation.

**Results:**

There was a significant decline in the MoCA score over time of −1.4 (95% CI −0.84 to −1.97) points over a 48-week period (*p* < 0.0001). Small but significant changes (*p* < 0.01) were observed in all MoCA domains except abstraction. The MoCA correlated weakly with the Repeatable Battery for the Assessment of Neuropsychological Status (RBANS) over time (*r*_rm_ = 0.1, *p* = 0.02) but exhibited a stronger correlation with the PSP Rating Scale (PSPRS) (*r*_rm_ = −0.25, *p* < 0.0001).

**Conclusion:**

The MoCA appears to have limited sensitivity in capturing cognitive decline in PSP.

## 1 Introduction

Progressive supranuclear palsy (PSP) is a form of atypical parkinsonism and tauopathy characterized by neuronal cell death in critical regions of the central nervous system such as the substantia nigra, basal ganglia, and subthalamic nucleus (1). Postural instability and ocular motor abnormalities are among the classic features of the disease. As part of their clinical presentation, PSP patients also frequently develop multi-domain cognitive dysfunction, including deficits in attention, memory, communication, visuospatial perception, and executive function (2). Accurate monitoring of these symptoms in clinical trial settings is crucial for determining the efficacy of pharmacological interventions.

The Repeatable Battery Assessment of Neuropsychological Status (RBANS) (3) is among the few cognitive assessments validated for use in PSP, assessing five critical domains: immediate memory, visuospatial/constructional ability, language, attention, and delayed recall. Notably, in longitudinal studies of participants with PSP, the RBANS has demonstrated clinically significant cognitive decline over 1 year as well as validity as a clinical trial outcome measure (4, 5).

The Montreal Cognitive Assessment (MoCA) (6) is a commonly used clinical scale for measuring cognitive impairment in clinical practice and clinical trials, including in PSP (7–9). Its brevity and ease of administration make it an attractive alternative to the RBANS. While RBANS administration takes approximately 20–30 min, MoCA takes roughly 10 min, with the time ranging based on the functional ability of the individual for both assessments. The MoCA comprises eight domains including visuospatial and executive functioning, naming, memory, attention, language, abstraction, delayed recall, and orientation, totaling 30 points. In North American populations, a score of 18–26 indicates mild cognitive impairment (MCI) (6), and a score of <18 is consistent with dementia in Parkinson’s disease (PD) (10), though this cutoff has not been specifically evaluated in PSP. Although initially developed as a screening tool, the MoCA has been used as a longitudinal measure of cognition across various neurological disorders such as MCI (11, 12), dementia (12), stroke (13–15) and ALS (16, 17). However, there are still conflicting reports about its sensitivity to detect progression of cognitive decline in related diseases like PD (18–22). Additionally, while an observational longitudinal study has reported on the change in MoCA over time (23), its utility as an outcome measure in clinical trials has yet to be studied. Here, we assess the longitudinal performance of the MoCA in a large well-characterized clinical trial cohort of participants with PSP.

## 2 Methods

All secondary analyses were performed in accordance with the ethical standards of Mass General Brigham Institutional Review Board in accordance with the Declaration of Helsinki. Written informed consent was obtained at the time of data collection and reconsent was not required for this secondary analysis.

Data were obtained from *N* = 162 members of the placebo arm of the Study of BIIB092 in Participants with Progressive Supranuclear Palsy (PASSPORT, clinicaltrials.gov identifier: NCT03068468), a phase 2, randomized, placebo-controlled trial evaluating the safety and efficacy of gosuranemab (a tau monoclonal antibody) in adults with PSP-Richardson syndrome (RS). Enrollment was limited to patients with probable or possible PSP based on the MDS PSP diagnosis criteria, symptom onset ≤5 years prior to baseline, aged 41–86. Patients with a score of ≤20 on the Mini-Mental State Examination or who were unable to ambulate independently were excluded. Participants were enrolled at 90 outpatient sites spanning 13 countries and underwent a 52-week double-blind phase, followed by an open-label extension period. Scores were initially recorded at baseline and then approximately every 12 weeks throughout the 124-week duration of the study (24). Owing to the high rate of dropout after week 52, we truncated the data after 52 weeks. Of note, the MoCA was performed at week 48 while the RBANS was performed at week 52. For the purposes of this analysis, we merged the data from these two time points.

An appropriate adaptation of all assessments, accounting for language and cultural relevance, was administered in each country that participated in the trial. Each domain of the RBANS yields scores ranging from 40 to 154, contributing to a total scaled score between 40 and 160 points (3), while each domain of the MoCA is scored on a scale of 0–6 points, with a maximum score of 30 points (6). Four parallel versions of the RBANS were administered sequentially every 3 months (A, B, C and D) resulting in a repetition of version A at week 52. MoCA version 7.1 was used repeatedly throughout the study.

All statistical analyses were performed using RStudio statistical software version 4.3.1. Linear mixed-effects models (LMM), fitted with the lme4 and lmerTest packages, were used to assess the temporal changes in the MoCA total and individual domain scores over the 52-week double-blind treatment period. LMM analysis is well-suited for longitudinal data as it can account for individual variability among subjects while effectively handling missing data under the missing at random (MAR) assumption (i.e., data missing dependent on data at hand), a frequent challenge encountered in longitudinal studies (25). Models included week (categorical), age at baseline, and sex as fixed effects with subject-specific random intercepts. Restricted Maximum Likelihood was used as the estimation method.

To begin to establish the criterion validity of MoCA as a repeated measures outcome, we evaluated its repeated measures correlations with those of the RBANS and PSPRS during the blinded period. We chose the RBANS as the gold standard assessment of cognitive function given its prior validation in PSP. The PSPRS was selected as the standard global PSP outcome measure for disease severity, which captures 6 domains (including ocular motor and limb motor, which can impact MoCA assessment) in addition to mentation (4 of 28 items, accounting for 16 of the 100 points) (26). We utilized an atypical application of ANCOVA (rmcorr function in R). This repeated measures correlation can account for the non-independence of repeated measures within individuals, making it especially fitting for longitudinal analyses. The rmcorr coefficient (r_rm_), ranging from −1 to 1, quantifies the strength of the linear association between the two variables. These effect sizes of the repeated measures correlation (r) can be interpreted as *r* = 0.1 small, *r* = 0.3 medium, *r* = 0.5 large (27).

## 3 Results

A total of 162 PSP patients were included in this analysis. Assessment data after week 52 were excluded due to the steep decline in participants after this timepoint. Demographic information and baseline scores for the MoCA, RBANS, PSPRS, and phonemic fluency are presented in Table 1. Longitudinal changes in these assessment scores are summarized in Supplementary Table 1. As shown in Supplementary Table 1, there was a decline in the number of participants who completed the MoCA from 155 at baseline to 133 at week 48, while participants who completed the RBANS declined from 152 at baseline to 113 at week 52 (compared to the 139 participants with PSPRS data at week 52).

**Table 1:** Baseline demographics and characteristics of participants in the placebo arm of the PASSPORT study.

Patient characteristics		*N*	*N* (%) or Mean (SD)
Age in years, mean (SD)		162	69.1 (6.7)
Sex, *N* (%)	Male	162	89 (55%)
	Female	162	73 (45%)
Race, *N* (%)	White	162	135 (83.3%)
	Other	162	24 (14.8%)
	Not listed	162	3 (1.9%)
MoCA total score, mean (SD)		155	21.7 (4.6)
RBANS total score, mean (SD)		152	74.9 (13.5)
RBANS Semantic Fluency score, mean (SD)		161	12.8 (4.0)
PSPRS total score, mean (SD)		162	37.2 (9.9)
Phonemic Fluency, mean (SD)		161	6.2 (3.8)

**Figure 1:**
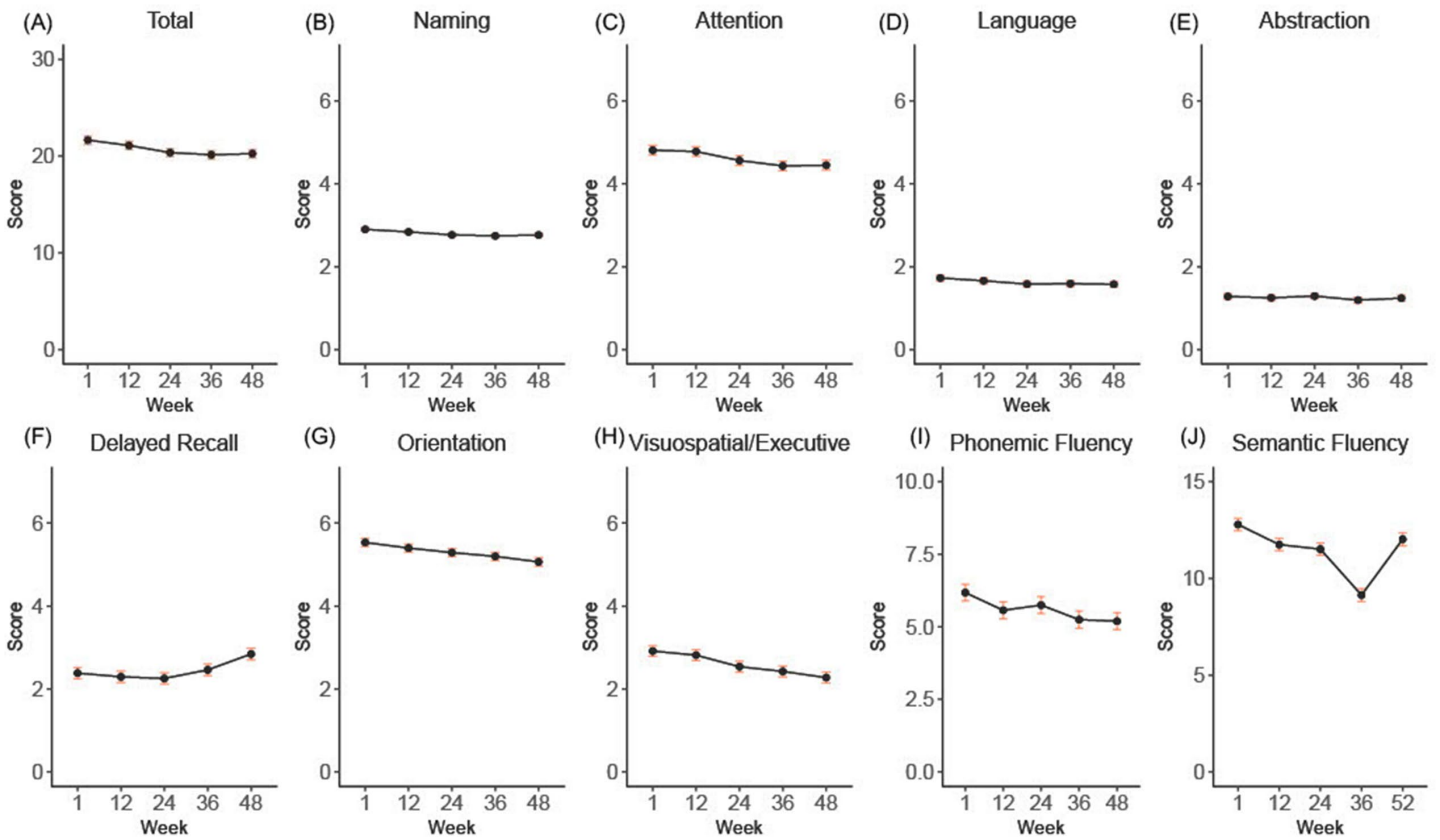
**(A-H)** shows the overall and subdomain progression of the total and subdomains of the Montreal Cognitive Assessment (MoCA) over the 52-week double-blind period. Panel **(I)** shows the raw phonemic fluency scores, and panel **(J)** shows the semantic fluency results extracted from the RBANS. Error bars indicate standard error.

LMM revealed a small but statistically significant time effect on the MoCA score (*p* < 0.0001). The adjusted mean MoCA score estimated at baseline was 21.7 with a 95% confidence interval (CI) of 20.8 to 22.5. Over the course of 48 weeks, a mean change of −1.4 (95% CI −0.84 to −1.97) points in MoCA scores was observed (Figure 1A).

To assess the contributions of each MoCA domain to the overall score decline over the 48-week period, we employed LMM for each individual domain (Figure 1B). Significant changes included: a decrease of −0.36 in attention (95% CI −0.16 to −0.56), an improvement of +0.46 in delayed recall (95% CI 0.20 to 0.72), a decrease of −0.15 in language (95% CI −0.03 to −0.28), a decrease of −0.14 in naming (95% CI −0.04 to − 0.23), a decrease of −0.47 in orientation (95% CI −0.29 to −0.65), a decrease of −0.64 in visuospatial/executive (95% CI −0.43 to −0.85), all *p* < 0.01. Abstraction decreased non-significantly by −0.04 (95% CI −0.16 to 0.07, *p* = 0.47).

To account for the binary manner in which the language data are captured with the MoCA, we also separately examined phonemic fluency, which was captured in a separately administered test, and semantic fluency, which was measured as part of the RBANS. From baseline to week 52, there was a statistically significant, although small decrease in mean phonemic fluency of −0.98 points (with a 95% confidence interval of −0.61 to −1.35, *p* < 0.0001) (Figure 1). In comparison, the semantic fluency sub score of the RBANS demonstrated a mean decline of −3.64 points (95% CI −3.07 to −4.22, *p* < 0.0001) by week 36, but increased 2.89 points by week 52 (95% CI 2.28 to 3.49), such that overall decline from baseline to week 52 was −0.76 (95% CI −0.17 to −1.35) (Figure 1).

**Figure 2:**
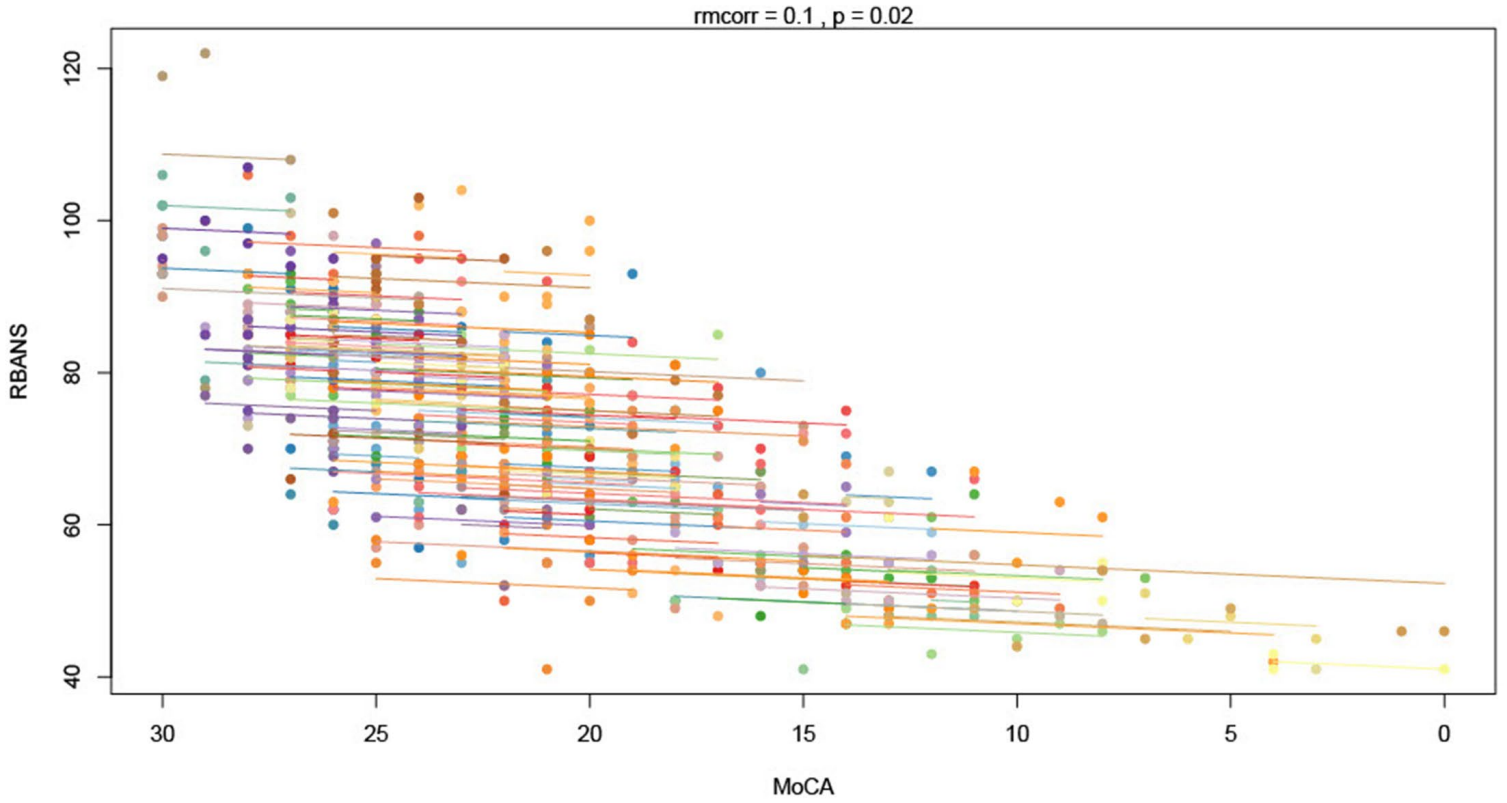
Repeated measures correlation (rmcorr) between the Montreal Cognitive Assessment (MoCA) and the RBANS. Each color indicates a distinct individual. Individual points demonstrate each time point for each individual and the overall slopes demonstrate the trajectory over time.

Our repeated measures correlation analysis revealed a weak correlation of the MoCA with the RBANS (*r*_rm_ = 0.1, *p* = 0.02, 95% CI 0.02 to 0.19) (Figure 2) and a modest correlation with the PSPRS (*r*_rm_ = −0.22, *p* < 0.0001, 95% CI −0.14 to −0.29) (Table 2). When evaluating the PSPRS subdomains individually, we found significant correlations between total MoCA scores and all domains except mentation and ocular motor. These results are summarized in Table 2.

**Table 2:** Repeated measures correlations between the MoCA and PSPRS domains.

PSPRS domain	r_rm_	*p*-value
History	−0.2	<0.0001
Mentation	−0.08	0.05
Bulbar	−0.18	<0.0001
Ocular motor	−0.08	0.04
Limb motor	−0.13	0.003
Gait and midline	−0.17	<0.0001
Total	−0.25	<0.0001

## 4 Discussion

The primary aim of our study was to assess the utility of the MoCA as a longitudinal clinical outcome measure in PSP. We found a statistically significant, although small, decline in MoCA scores over a 52-week period in all domains, except for abstraction and recall. Delayed recall improved slightly in participants, likely demonstrating practice effects. Overall, the changes seem to have limited clinical significance. The increase in semantic fluency scores from week 36 to 52 may have also been related to practice effects.

The repeated measures correlations between MoCA scores and those of the RBANS and PSPRS revealed only weak associations over time. Interestingly, a slightly stronger correlation was identified between the MoCA and PSPRS than between the MoCA and the RBANS. This suggests that the MoCA may reflect other deficits of PSP, as opposed to providing a pure assessment of cognition/cognitive domains; this has been observed in other cognitive outcome measures, including the RBANS (28).

In particular, the visuospatial domain of the MoCA requires ocular motor and motor function for the drawing tasks. Previous research by Jaegar et al. has attempted to address this by developing a cognitive composite battery for PSP that accounts for the confounding effects of motor impairment (29). The lack of correlation with the mentation domain of the PSPRS suggests that the mentation domain may be limited in its ability to capture cognitive impairment in PSP across multiple time points. It may also be due to the fact that the mentation domain of the PSPRS captures aspects of mood and behavior, which the MoCA does not.

Our study had several limitations. First, we chose the RBANS as the comparator standard for cognitive function and decline because few cognitive instruments have been validated in PSP (30). However, there are limitations to the RBANS as well, such as the length of administration and limited sensitivity. Second, the inherent limitations of the rmcorr function in R in handling missing data restricted our analysis to participants with no missed visits, and our findings should be interpreted within the context of the limited dataset. Third, using the same version of the MoCA every time likely resulted in a practice effect that contributed to the small decrease in scores seen over time (and the improved delayed recall and semantic fluency tests at the end of the study). Lastly, while the LMM analysis did account for missing data under the MAR assumption, it is likely that the missing data may not have been random (i.e., participants with steeper cognitive decline being more likely to drop out of the study), which could contribute to the surprising apparent lack of decline in the MoCA over time. Examining only participants who discontinued the study before week 52, the mean MoCA score at baseline was 20.8 (*n* = 19) which is slightly lower than the average of the other participants. While the sample size is limited, this suggests that missingness was not completely at random.

Overall, our results are in line with a previous observational cohort study including all PSP variants that found an annual rate of progression of −0.9 points in the MoCA score (SD 3.7, *n* = 117). In the RS subgroup, the rate was −0.8 (SD 3.0, *n* = 57) (23). While the MoCA was able to capture the cognitive impairment that is common in PSP (average baseline MoCA scores were < 26), the changes observed over the one-year period were surprisingly small in magnitude, showing a lack of sensitivity in capturing the cognitive decline associated with PSP as revealed by the RBANS. These preliminary findings show that the MoCA is insensitive to the natural progression of the disease and cast doubt on its use as a cognitive outcome measure in PSP. However, this does not completely rule out the possibility of the MoCA showing improvement over time in response to a symptomatic treatment. Altogether, this suggests that the MoCA suggests that should only be used to screen for cognitive impairment. These findings underscore the need for more specific and sensitive instruments to comprehensively and conveniently evaluate cognitive function over time in PSP.

## Data Availability

Publicly available datasets were analyzed in this study. This data can be found here: https://portal.rdca.c-path.org/.
