# Supplementary material for: Montreal cognitive assessment as a cognitive outcome measure in progressive supranuclear palsy
Source: Front Neurol. 2024 Dec 4;15:1501206. doi: 10.3389/fneur.2024.1501206 (PMC11704677; doi:10.3389/fneur.2024.1501206)
Supplement: Supplementary file 1 [file Table_1.docx]

**Supplementary Table 1**

| **Assessment** |  | **Week 12** | **Week 24** | **Week 36** | **Week 48** | **Week 52** |
| --- | --- | --- | --- | --- | --- | --- |
| MoCA | N | 151 | 150 | 141 | 131 | ---- |
|  | Mean (SD) | 21.1 (5.5) | 20.5 (5.6) | 20.1 (6.4) | 20.3 (6.7) | ---- |
| RBANS | N | 144 | 134 | 125 | ---- | 113 |
|  | Mean (SD) | 70.3 (13.9) | 73.1 (15.3) | 71.5 (14.7) | ---- | 70.9 (14.9) |
| PSPRS | N | 159 | 155 | 147 | 139 | 139 |
|  | Mean (SD) | 39.5 (11.1) | 41.6 (11.9) | 43.8 (13.1) | 45.3 (13.4) | 47.2(14.0) |
| Phonemic Fluency | N | 157 | 150 | 145 | 138 | ---- |
|  | Mean (SD) | 5.5 (3.7) | 5.8 (3.9) | 5.3 (3.6) | 5.3 (3.7) | ---- |
| RBANS Semantic Fluency | N | 155 | 151 | 143 | ---- | 135 |
|  | Mean (SD) | 11.7 (3.6) | 11.6 (4.3) | 9.2 (4.9) | ---- | 12.1 (4.3) |

Supplementary Table 1: Mean (SD) assessment scores of participants in the placebo arm over the remainder of the 52-week study. The number of remaining participants at each time point is included.
